# Spontaneous breathing trial with pressure support on positive end-expiratory pressure and extensive use of non-invasive ventilation versus T-piece in difficult-to-wean patients from mechanical ventilation: a randomized controlled trial

**DOI:** 10.1186/s13613-024-01290-6

**Published:** 2024-04-17

**Authors:** Mehdi Mezidi, Hodane Yonis, Louis Chauvelot, Guillaume Deniel, François Dhelft, Maxime Gaillet, Ines Noirot, Laure Folliet, Paul Chabert, Guillaume David, William Danjou, Loredana Baboi, Clotilde Bettinger, Pauline Bernon, Mehdi Girard, Judith Provoost, Alwin Bazzani, Laurent Bitker, Jean-Christophe Richard

**Affiliations:** 1 Medical Intensive Care Unit, Croix-Rousse Hospital, Hospices Civils de Lyon, Lyon, France; 2 Université Lyon 1, Université de Lyon, Lyon, France; 3 CREATIS INSERM, 1044 CNRS 5220, Villeurbanne, France

**Keywords:** Pressure support, Spontaneous breathing trial, T-piece, Positive end-expiratory pressure, Difficult weaning

## Abstract

**Background:**

The aim of this study is to assess whether a strategy combining spontaneous breathing trial (SBT) with both pressure support (PS) and positive end-expiratory pressure (PEEP) and extended use of post-extubation non-invasive ventilation (NIV) (extensively-assisted weaning) would shorten the time until successful extubation as compared with SBT with T-piece (TP) and post-extubation NIV performed in selected patients as advocated by guidelines (standard weaning), in difficult-to-wean patients from mechanical ventilation.

**Methods:**

The study is a single-center prospective open label, randomized controlled superiority trial with two parallel groups and balanced randomization with a 1:1 ratio. Eligible patients were intubated patients mechanically ventilated for more than 24 h who failed their first SBT using TP. In the extensively-assisted weaning group, SBT was performed with PS (7 cmH_2_O) and PEEP (5 cmH_2_O). In case of SBT success, an additional SBT with TP was performed. Failure of this SBT-TP was an additional criterion for post-extubation NIV in this group in addition to other recommended criteria. In the standard weaning group, SBT was performed with TP, and NIV was performed according to international guidelines. The primary outcome criterion was the time between inclusion and successful extubation evaluated with a Cox model with adjustment on randomization strata.

**Results:**

From May 2019 to March 2023, 98 patients were included and randomized in the study (49 in each group). Four patients were excluded from the intention-to-treat population (2 in both groups); therefore, 47 patients were analyzed in each group. The extensively-assisted weaning group had a higher median age (68 [58–73] vs. 62 [55–71] yrs.) and similar sex ratio (62% male vs. 57%). Time until successful extubation was not significantly different between extensively-assisted and standard weaning groups (median, 172 [50–436] vs. 95 [47–232] hours, Cox hazard ratio for successful extubation, 0.88 [95% confidence interval: 0.55–1.42] using the standard weaning group as a reference; *p* = 0.60). All secondary outcomes were not significantly different between groups.

**Conclusion:**

An extensively-assisted weaning strategy did not lead to a shorter time to successful extubation than a standard weaning strategy.

*Trial registration* The trial was registered on ClinicalTrials.gov (NCT03861117), on March 1, 2019, before the inclusion of the first patient. https://clinicaltrials.gov/study/NCT03861117.

**Supplementary Information:**

The online version contains supplementary material available at 10.1186/s13613-024-01290-6.

## Background

Approximately 40% of patients will receive invasive mechanical ventilation during their intensive care unit (ICU) stay [1]. During the mechanical ventilation weaning process, the patient’s ability to breathe without mechanical support is usually assessed with spontaneous breathing trials (SBTs) to mimic post-extubation work of breathing. SBT with pressure support (SBT-PS) and SBT with T-piece (SBT-TP) are the two main modalities used in ICUs worldwide [1]. The SBT-PS consists in lowering the pressure support level with or without positive end expiratory pressure (PEEP), while the SBT-TP consists in disconnecting the patient from the ventilator and connecting a T-piece to administer supplemental oxygen if needed. The theoretical benefits of SBT-PS over SBT-TP are related to a lower work of breathing compared to the former, which could subsequently promote earlier extubation [2], and prevent weaning-induced pulmonary oedema (WIPO), more so if PEEP is added to PS [3]. The 2017 ATS guidelines [4], based on the meta-analysis of 3 randomized controlled trials available at this time, suggested that the initial SBT should be conducted with inspiratory pressure augmentation (5–8 cm H_2_O) rather than without (T-piece or continuous positive airway pressure) in patients ventilated for more than 24 h, as both SBT success and extubation success were significantly higher with SBT-PS.

In a population of ICU patients with a high probability of weaning success, a large randomized controlled trial has recently shown that a short session of 30 min with SBT-PS with zero end-expiratory pressure (ZEEP) was also associated with a significantly higher rate of successful extubation than a longer session lasting 2 h with SBT-TP [5]. However, in patients at *high risk* of extubation failure, another recent randomized controlled trial failed to demonstrate that SBT-PS with ZEEP reduced ventilator-free days at day-28 compared to SBT-TP [6], although the rates of successful first SBT and extubation success within 24 h were significantly higher with SBT-PS. Whether these results apply to difficult-to-wean patients remains unknown, and it is possible that promoting earlier extubation with inspiratory pressure augmentation in this subgroup of patients might increase the rate of extubation failure and the rate of WIPO during the transition from positive pressure to atmospheric pressure after extubation.

Hence, we hypothesized that the combination of PEEP and PS during SBT and extended use of post-extubation NIV to prevent WIPO (the extensively-assisted weaning strategy) may shorten the time until successful extubation compared with T-piece and post-extubation NIV performed only in patients at high risk of extubation failure based on their comorbidities or hypercapnia during the spontaneous breathing trial [4] (the standard weaning strategy). We further hypothesized that failure of an SBT-TP after a successful SBT-PS would better identify patients at high risk of extubation failure who would benefit from prophylactic post-extubation NIV to avoid reintubation.

## Methods

### Trial design

The study was a monocentric prospective open label, randomized controlled superiority trial with 2 parallel groups and balanced randomization with a 1:1 ratio. The trial protocol was previously published [7]. The study was conducted in one ICU located in a French academic hospital and was registered at clinicalTrials.gov (NCT03861117) before the inclusion of the first patient. The study was approved by a research ethics committee (CPP Ile-de-France VI) under IDRCB 2019-A00106-51. The trial protocol was amended to prolong the recruitment period and to allow consent of patients unable to write through a third-party attestation. Written consent from the patient (or next of kin) was obtained for all patients.

### Patients

Adult patients who were intubated and mechanically ventilated for more than 24 h with weaning readiness criteria (Additional file 1) and who failed a first SBT-TP were eligible. Non-inclusion criteria are presented in Additional file 2. The intention-to-treat population consisted of all randomized subjects, with the exception of patients excluded from the study for the following reasons: transfer to another ICU, consent withdrawal by patient or next of kin, or erroneous inclusion without eligibility criteria.

### Randomization and Interventions

Patients were randomly assigned (1:1) to the extensively-assisted weaning group or the standard weaning group. The allocation sequence was computer-generated with stratification into 3 strata: patients with chronic left ventricular systolic heart failure (CHF) defined by a left ventricular ejection fraction < 45%, patients with suspected or proven chronic obstructive pulmonary disease (COPD) [8] and patients without CHF or COPD. In the case of concomitant CHF and COPD, patients were stratified in the COPD strata. Randomization was performed in each stratum using random block sizes of 4, 6 and 8. Allocation sequences were concealed in sealed opaque envelopes by a clinical research assistant who did not participate in the assessment of patient eligibility for the study.

The interventions were started within the 6 h following inclusion, with the execution of the allocated SBT, and are summarized in Additional file 3.

#### Procedures common to both groups

While still intubated and presenting weaning readiness criteria (Additional file 1) [4, 9, 10], patients underwent a daily SBT for 30 min with the allocated SBT. SBT success was evaluated by nurses and defined by the ability to perform the SBT for 30 min without SBT failure criteria (Additional file 4). In case of SBT failure, patients were switched back to previous respiratory parameters. The cough strength and abundancy of respiratory secretions were quantified by the attending physician using ordinal scores [11, 12] (Additional file 5), and readiness to extubate criteria were assessed (Additional file 6). Patients meeting weaning and readiness to extubate criteria were extubated 2 to 3 h after resuming ventilation with prior ventilator settings and received post-extubation NIV if they met at least one of the following post-extubation prophylactic NIV criteria [4]: age > 65 yrs., CHF, CRF, COPD or carbon dioxide partial pressure in arterial blood (PaCO_2_) > 45 mmHg at the end of the SBT.

Post-extubation prophylactic NIV was performed per sessions of 1 to 2 h every 3 h, with a minimum of 8 h per day, for at least 24 h following extubation or until reintubation, whichever occurred first.

Post-extubation rescue NIV for respiratory failure was allowed only in case of suspicion of WIPO or hypercapnic respiratory failure for COPD/CRF patients. Reintubation criteria are provided in Additional file 7. In case of reintubation, the patients remained treated with their allocated strategy until successful extubation, death or day-90 from inclusion, whichever came first. In case of self-extubation, patients were managed similarly to patients with scheduled extubation. In patients with a decision of tracheostomy after study inclusion, weaning management according to study allocation ceased, but the subjects were analyzed according to their allocated group.

#### Procedures specific to the extensively-assisted weaning group

The daily SBT was done with pressure support 7 cmH_2_O (SBT-PS), PEEP 5 cmH_2_O and inspired oxygen fraction (FiO_2_) set between 21 and 50% to target a peripheral oxygen saturation (SpO_2_) between 94 and 98% (or between 88 and 92% for COPD and chronic respiratory failure (CRF) patients). In case of SBT-PS success, an additional SBT-TP was performed after 30 min of rest under prior ventilator settings, for a maximal duration of 30 min. In case of SBT-PS success, an additional SBT-TP was performed after 30 min of rest under prior ventilator settings for a maximal duration of 30 min. The modalities of this additional SBT-TP were the same as those in the control group (see below). In case of additional SBT-TP failure, patients were extubated if they met the readiness to extubate criteria but received post-extubation prophylactic NIV with the same modalities as above.

#### Procedures specific to the standard weaning group

The daily 30-min SBT was done by disconnecting the patient from the ventilator and using a T-piece to administer oxygen targeting an SpO_2_ between 94 and 98% (or between 88 and 92% for COPD/CRF patients).

### Outcomes

#### Primary outcome

The primary outcome was the time between study inclusion and time of extubation [or cessation of ventilatory support in tracheotomized patients] in patients without reintubation or death within the 7 days following extubation. In patients not meeting extubation success criteria, data were censored on day-90 or date of death, whichever came first. Reintubation and resuming mechanical ventilation for less than 24 h related to unplanned short procedures were not considered as extubation failures. Patients extubated and discharged from the study ICU alive within the 7 days following extubation were considered successful extubation.

#### Secondary outcomes

Prespecified secondary outcomes were the following: rate of successful extubation on the first extubation attempt; invasive mechanical ventilation duration (after inclusion); mechanical (invasive and non-invasive) ventilation duration after inclusion; ventilator-free days at day-28 and day-90; ICU and hospital length of stay after inclusion; ICU, day-28 and day-90 mortality; and reintubation rate. Minor modifications occurred to some secondary outcomes after trial onset and before database lock to improve reproducibility with comparable studies [7]. Computation of reintubation rate was modified and post hoc additional outcomes were added during the reviewing process (Additional file 8).

#### Sensitivity analyses

Prespecified sensitivity analyses were performed on the primary outcome criterion in the following subgroups: 1- randomization strata; 2- COVID-19 status at inclusion; 3- serum bicarbonate level on the day of extubation below vs. greater or equal to its median value; 4- PaCO_2_ at the end of SBT ≥ 45 mmHg vs. < 45 mmHg, on the day of extubation; 5- time between intubation and inclusion below vs. greater or equal to its median value; and 6- cough score below vs. greater or equal to its median value.

### Blinding

Due to the type of intervention, clinicians and patients were not blinded to group allocation. Data analysts were blinded to group allocation.

### Data collection

Baseline data were collected at ICU admission and at study inclusion. Daily weight was used to compute cumulative fluid balance between ICU admission and study inclusion. After inclusion, daily assessment of weaning-readiness criteria, SBT results, and readiness to extubate criteria were collected, and in case of extubation, characteristics of extubation, post-extubation care and reintubation episodes were recorded. We retrospectively checked for the presence of a high risk of extubation failure on the day of extubation if ≥ 4 risk factors were present (Additional file 9) [13].

### Sample size

In a pilot observational study on 88 patients in our center, the median time between the first SBT and extubation was 22 h with a strategy using SBT-PS and PEEP. We hypothesized that the time until successful extubation in the SBT-TP would be 24 h longer. With an alpha risk of 5% and a bilateral hypothesis, a power of 80% and a hazard ratio (HR) of 2 (based on the ratio of median duration of time between first SBT and successful extubation in each group), a total of 66 events would be needed. Accounting for both potential deaths before successful weaning and weaning failure (assumed to amount to 30%), we planned to include 94 patients (47 per group).

### Statistical analysis

Quantitative variables were described using the median [interquartile range (IQR)] or mean (standard deviation [SD]). Hodges‒Lehmann method was used to compute unbiased medians of the between-group differences and their 95% confidence intervals (CI_95%_). Qualitative variables were reported as numbers (absolute frequencies). Absolute rate differences between groups were provided, along with their CI_95%_ computed with bootstrapping. Missing data number were reported per variable.

Time to successful extubation was analyzed according to a cause-specific Cox model with randomization strata as a covariate. To account for the competing risk of death, a sensitivity analysis was performed on the main outcome using a Fine and Gray model with successful extubation as event and death as competitive risk, with randomization strata as covariate. Unplanned post hoc analyses are specified in Additional file 10.

Subgroup sensitivity analysis was performed through a Cox model with time to successful extubation as the dependent variable and a multiplicative interaction term (randomization group*subgroup variable) as the explanatory variable for each subgroup variable.

A *p-*value < 0.05 with a bilateral hypothesis was considered significant. All analyses were carried out using R for Mac with forestploter, ggplot2, cmprsk, and survival packages [14–18].

## Results

### Patients

**Fig. 1:**
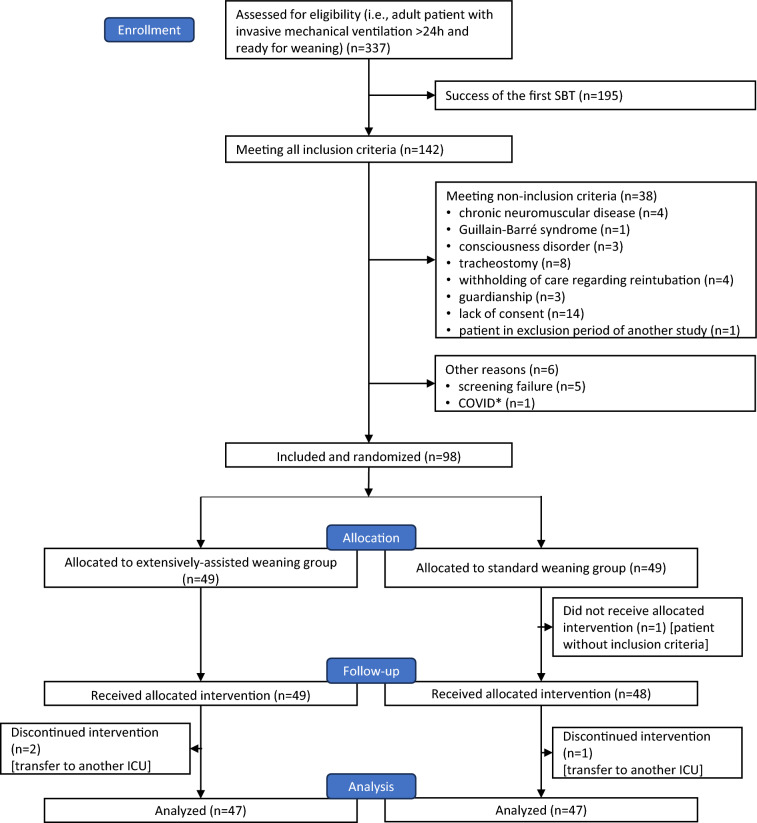
Flow chart of the study; *ICU* intensive care unit; *pts* patients. *One patient was not included at the beginning of the COVID-19 pandemic due to lack of data regarding the risk of COVID-19 transmission to healthcare workers during T-piece trials. Inclusions were then transiently interrupted (see text for details)

From May 2019 to March 2023, 98 patients were included and randomized in the study (Fig. 1). Inclusions were suspended for 6 months due to the COVID-19 outbreak. Four patients were excluded from the intention-to-treat analysis (2 in each group), and the intention-to-treat population consisted of 94 patients (47 patients in each group). The median [IQR] time between the failed SBT used for study eligibility and study inclusion was 3 [1–6] h.

Baseline characteristics are reported in Table 1. Patients in the extensively-assisted weaning group were older (68 [58–73] vs. 62 [55–71] years) than patients in the standard weaning group, had higher simplified acute physiology score II (SAPS2) scores at ICU admission (57 (15) vs. 50 (16)), and were more frequently under volume assist-control ventilation at inclusion (81% vs. 47%).

**Table 1:** Baseline characteristics at inclusion

Variables	Extensively-assisted weaning group (n = 47)	Standard weaning group (n = 47)
Median age [IQR]—yr	68 [58–73]	62 [55–71]
Male sex—no. (%)	29 (62%)	27 (57%)
Mean body mass index (SD)—kg/m^2^	28.1 (7.1)	29.9 (8.3)
Median Charlson score [IQR]	4 [3–6]	3 [2–5]
Mean SAPS2 score at ICU admission (SD)	57 (15)	50 (16)
Cause of ICU admission—no. (%)		
Planned surgery	2 (4%)	5 (11%)
Emergent surgery	0 (0%)	4 (9%)
Medical	45 (96%)	38 (81%)
Cause(s) for intubation—no. (%)		
Hemodynamic	5 (11%)	6 (13%)
Neurologic	6 (13%)	8 (17%)
Post-operative	1 (2%)	5 (11%)
Respiratory	45 (96%)	38 (81%)
COPD		
Proven—no. (%)	11 (23%)	8 (17%)
Suspected—no. (%)	3 (6%)	3 (6%)
Chronic respiratory failure—no. (%)	10 (21%)	7 (15%)
Home respiratory support		
Oxygen without NIV—no. (%)	3 (6%)	1 (2%)
NIV with or without oxygen– no. (%)	6 (13%)	5 (11%)
Chronic heart failure—no. (%)	6 (13%)	5 (11%)
COVID-19—no. (%)	17 (36%)	18 (38%)
Median time between intubation and inclusion [IQR]—days	7 [4–20]	8 [4–22]
Mean SOFA score (SD)	7 (3)	6 (3)
Randomization strata		
COPD—no. (%)	14 (30%)	11 (23%)
CHF—no. (%)	4 (9%)	4 (9%)
No COPD nor CHF—no. (%)	29 (62%)	32 (68%)
Ventilatory mode		
Volume assist-control ventilation—no. (%)	38 (81%)	22 (47%)
Pressure support—no. (%)	9 (19%)	25 (53%)
Median FiO_2_ [IQR]—%	35 [30–40]	30 [30–40]
Mean tidal volume (SD)—mL	378 (120)	389 (107)
Mean pressure support level (SD)—cmH_2_O*	11 (4)	10 (3)
Median positive end-expiratory pressure [IQR]—cmH_2_O	5 [5–5]	5 [5–5]
Mean respiratory rate (SD)—/min	25 (6)	24 (5)
Mean minute-ventilation (SD)—L/min	9.5 (3.1)	9.3 (3.1)
Mean pH (SD)**	7.45 (0.07)	7.45 (0.06)
Mean PaO_2_ (SD)—Torr**	80 (23)	76 (13)
Mean PaCO_2_ (SD)—Torr**	45 (10)	44 (9)
Median lactate [IQR]—mmol/L†	2 [1, 2]	2 [1, 2]
Mean cumulative fluid balance (SD)—kg^‡,§^	− 3.2 (8.4)	− 5.6 (9.5)

CHF denotes chronic heart failure; COPD, chronic obstructive pulmonary disease; CRF, chronic respiratory failure; IQR, interquartile range; SD, standard deviation; SAPS2, simplified acute physiology score II; ICU, intensive care unit; FiO_2_, inspired fraction of oxygen; NIV, non-invasive ventilation; PaO_2_, partial pressure of arterial oxygen; PaCO_2_, partial pressure of arterial carbon dioxide; and SOFA, sequential organ failure assessment score

Variables are expressed as median [IQR] when their distribution is asymmetrical, or mean (SD) otherwise

^*^Among patients in pressure support mode (extensively-assisted weaning group: 9, standard weaning group: 25)

^**^Blood gases parameters at inclusion were missing for 1 patient from the standard weaning group

^†^Lactate was missing for 8 patients in the extensively-assisted weaning group and 6 in the standard weaning group

^‡^between ICU admission and inclusion

^§^Cumulative fluid balance at inclusion was missing for 2 patients in the extensively-assisted weaning group and 1 in the standard weaning group

### Outcomes

**Fig. 2:**
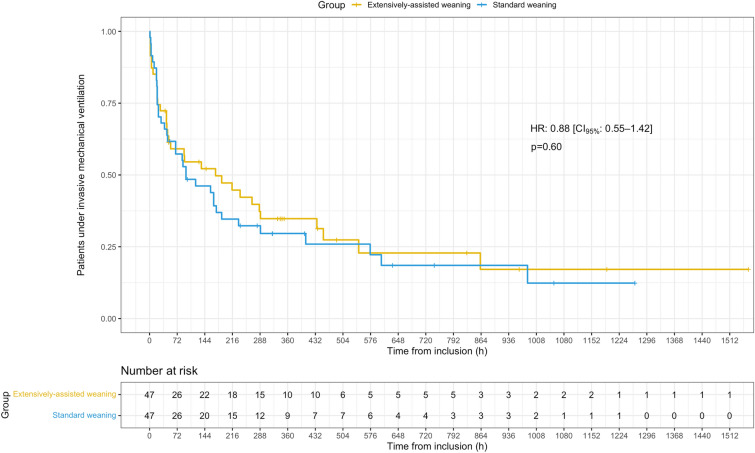
Kaplan‒Meier curve depicting the time to successful extubation. HR denotes hazard ratio and CI_95%_, 95% confidence interval

One patient of the extensively-assisted weaning group was tracheotomized after inclusion. Time until successful extubation was not significantly different between groups (172 [50–436] vs. 95 [47–232] hours in the extensively-assisted and standard weaning groups, respectively), with a HR for successful extubation of 0.88 [CI_95%_: 0.55–1.42] (*p* = 0.60) using the standard weaning group as a reference (Fig. 2, Table 2). Competitive risk analysis with the Fine and Gray model (Additional file 11, Table 2) led to similar results (subdistribution hazard ratio (SHR) for successful extubation: 0.84 [CI_95%_: 0.53–1.34], *p* = 0.47). All secondary outcomes were also similar between groups (Table 2). The median time to the first extubation attempt was 44 [20–56] in the extensively-assisted weaning group vs. 50 [38–95] hours in the standard weaning group (Additional file 12, post hoc analysis),* p* = 0.50. Prespecified sensitivity analyses identified no significant interaction regarding the primary judgment criterion. Additional outcomes are reported in Table 3, and were not significantly different between groups.

**Table 2:** Primary and secondary outcomes

Variables	Extensively-assisted weaning (n = 47)	Standard weaning (n = 47)		*p-value*
	Median [CI_95%_]	Median [CI_95%_]	HR or SHR [95%CI]	
Primary outcome				
Time until successful extubation—Cox model—hrs. §	172 [50–436]	95 [47–232]	0.88 [0.55–1.42]	0.60
Secondary outcomes				
Time to successful extubation—Fine and Gray model—hrs. §§	172 [50–436]	95 [47–232]	0.84 [0.53–1.34]	0.47
Time to death—Fine and Gray model—hrs. §§	488 [346–NR]	633 [321–NR]	1.30 [0.58–2.93]	0.53

	Median [IQR]	Median [IQR]	Median difference [CI_95%_]*	*p*-*value*
Invasive mechanical ventilation duration [IQR]—hrs. †	129 [25–344]	95 [22–285]	5 [-37–68]	0.73
Mechanical ventilation duration [IQR]—hrs. †	187 [61–355]	122 [46–321]	25 [-42–97]	0.34
Median ventilator-free days at day-28 [IQR]—day †	16 [0–26]	20 [0–26]	0 [− 3–1]	0.59
Median ventilator-free days at day-90 [IQR]—day †	77 [0–88]	82 [0–88]	0 [− 2–1]	0.72
Median ICU length of stay [IQR]—day †	8 [4–18]	9 [4–16]	0 [− 3–3]	0.84
Median hospital length of stay [IQR]—day †	25 [15–47]	26 [12–65]	0 [− 10–8]	0.94

	No. (%)	No. (%)	Absolute rate difference [CI_95%_]*	*p-value*
Rate of successful extubation on the first extubation attempt	22 (47%)	27 (57%)	− 11 [− 30 –11]%	0.41
ICU mortality	14 (30%)	13 (28%)	2 [− 17–21]%	1
Day-28 mortality	15 (32%)	12 (26%)	6 [− 13–23]%	0.65
Day-90 mortality	19 (40%)	15 (32%)	8 [− 11–28]%	0.52
Rate of patients experiencing reintubation among extubated patients	18/41 (44%)	17/44 (39%)	5 [− 16–26]%	0.66

^§^Cox model including randomization group and strata as explanatory variables

^§§^Fine and Gray model including randomization group and strata as explanatory variables

CI_95%_ denotes 95% confidence interval; HR, hazard ratio; SHR, subdistribution hazard ratio; NR, not reached; IQR, interquartile range andICU, intensive care unit

^*^Median differences with their CI_95%_ were computed with the Hodges Lehmann method and absolute rate difference and CI_95%_ were computed for proportions using bootstrapping

^†^Since inclusion

**Table 3:** Additional outcomes added during the reviewing process

Variables	Extensively-assisted weaning group (n = 47)	Standard weaning group (n = 47)	Absolute rate difference [CI_95%_]*	*p-value*
Rate of successful first SBT after inclusion—No. (%)	16 (34%)	9 (19%)	15 [− 2–32]%	0.16
Rate of successful extubation on day-1 after the first SBT—No. (%)	7 (15%)	5 (11%)	4 [− 9–17]%	0.76
Rate of successful extubation within 7 days after inclusion—No. (%)	22 (47%)	28 (60%)	− 13 [− 32–6]%	0.30
Rate of patients experiencing an extubation attempt—No. (%)	41 (87%)	44 (94%)	− 6 [− 19–4]%	0.49
Rate of patients with a self extubation among extubated patients—No. (%)	6/41 (15%)	2/44 (5%)	10 [− 2–22]%	0.15

CI_95%_ denotes 95% confidence interval; pts, patients; and SBT, spontaneous breathing trial

^*^Absolute rate difference and CI_95%_ were computed for proportions using bootstrapping

### Extubation success and failure

Eighty-five patients had at least one extubation attempt (41 in the extensively-assisted weaning group and 44 in the standard weaning group, Additional file 13, post hoc analysis). The rate of extubation attempts with prophylactic post-extubation NIV was not significantly different between groups (56/72 (78%) in the extensively-assisted weaning group vs. 43/61 (70%) in the standard weaning group,* p* = 0.43). Extubation episodes with a high risk of extubation failure amounted to 65/72 (90%) and 54/61 (89%) of all extubation attempts in the extensively-assisted weaning and standard weaning groups, respectively (absolute difference: 2 [CI_95%_ − 9–12]%,* p* = 0.78).

The rate of successful extubation was similar between patients succeeding their allocated SBT (n = 31/60 [52%] in the extensively-assisted weaning group and n = 36/57 [63%] in the standard weaning group, absolute difference − 11 [− 29–6]%, *p* = 0.24). Among patients in the extensively-assisted weaning succeeding their allocated SBT with an unsuccessful additional TP trial, rate of successful extubation was 44% (n = 8/18) (Additional file 14, post hoc analysis).

The rate of patients experiencing reintubation amounted to 18/41 (44%) and 17/44 (39%) in the extensively-assisted and standard weaning groups respectively (absolute difference: 5 [CI_95%_ − 16–26]%, *p* = 0.66, Table 2 and Additional file 15). The main reason for reintubation was respiratory failure in both groups (92% and 86% in the extensively-assisted and standard weaning groups, respectively). The median time between extubation and reintubation was not significantly different in the extensively-assisted weaning group 7 [2–49] vs. 18 [7–61] hours in the standard weaning group (absolute difference: − 5 [CI_95%_ − 18–3] hours, *p* = 0.20, post hoc analysis).

Thirty-eight patients (81%) in each group were retrospectively classified as difficult or prolonged weaning according to the WIND classification [1] (Additional file 16, post hoc analysis), while the remaining patients were extubated within 24 h of the first failed separation attempt from the ventilator and classified as simple weaning.

### Protocol adherence

The rate of protocol violation was similar in both groups. However, in the standard weaning group, there was a trend toward more frequent violations due to post-extubation NIV performed despite not required per protocol (Additional file 17). On another hand, the additional SBT-TP was not performed in the extensively-assisted weaning group in 13/47 (28%) patients.

## Discussion

In this single-center randomized controlled trial, an extensively-assisted weaning strategy compared to a standard weaning strategy did not result in a shorter time to successful extubation. Furthermore, this strategy was associated with a non-significant decrease in time to first extubation and a nonsignificant increase in the reintubation rate. Finally, by combining two subsequent SBTs with PS and TP, we were able to identify a subgroup of patients with a high rate of extubation failure of approximately 60% (i.e., in patients with successful SBT-PS and unsuccessful SBT-TP).

The study population is singular, as 81% of the patients were classified as presenting a difficult or prolonged weaning according to the WIND definition, while this population represented 24% of the patients entered in the weaning process in the WIND study [1]. As a consequence, ICU mortality was substantial but similar to patients belonging to the prolonged weaning group of the WIND study (i.e., 30%) [1]. A high rate of reintubation was observed in both groups of the present study, and this may be related to the 90% rate of patients deemed at high risk of extubation failure [13]. This high reintubation rate is however not unexpected, since Béduneau et al. identified a 45% reintubation rate in the WIND study in the group of patients presenting with difficult or prolonged weaning [1]. Discrepancies exist in the literature regarding reintubation rates in patients with difficult or prolonged weaning. A lower reintubation rate (i.e., 10%) was recently observed in a recent large observational study in patients who failed their first SBT, but in a population with substantially lower mortality and a lower rate of respiratory failure as reason for intubation [19]. In the WEAN SAFE study, patients undergoing difficult or prolonged weaning had reintubation rates of 32% and 72%, respectively [20]. These variations may stem from differences in case mix, but also from discrepancies in defining difficult or prolonged weaning—whether by the result of the initial SBT or the time between the initial SBT and successful extubation—variances in use of tracheostomy following initial SBT failure,—or variation in the delay to initiate the weaning process [20].

Finally, the present study population differs from virtually all previously published randomized controlled trials on intubated patients, as they did not specifically include patients failing their first SBT [5, 6, 21–23], or compared daily SBT-TP vs. gradual reduction of pressure support in difficult to-wean patients [24, 25].

The lack of superiority of SBT-PS over SBT-TP for patients with difficult weaning was not expected. Cabello [2] demonstrated that in difficult-to-wean patients, SBT-TP generated a work of breathing twice as high as SBT-PS, making the latter an “easier” test. Our results differ from the results of two recent large randomized controlled trials [5, 6] in which SBT-PS was associated with a higher rate of successful extubation than SBT-TP. In addition to differences regarding inclusion of patients failing their first SBT and realization of SBT-PS with PEEP as opposed to ZEEP, our study differs from both studies by a substantially higher rate of respiratory failure as the main reason for intubation and a longer time between intubation and inclusion. Another explanation for this difference in outcome may be related to a slight imbalance at randomization in our study regarding age and SAPS2 disfavoring the SBT-PS group. Finally, the lack of difference between both groups might also be explained by a greater use of out-of-protocol post-extubation NIV in the standard weaning group that may have prevented extubation failure in this group.

While the time to first extubation was nonsignificantly shorter in the extensively-assisted weaning group, the time to successful extubation was nonsignificantly higher in this group and was associated with a nonsignificant increase in the reintubation rate. Taken together, these findings suggest that SBT-PS with additional PEEP, by providing excessive respiratory assistance during the SBT, could have driven toward an anticipated extubation before resolution of the primary cause of intubation. It is therefore plausible that patients extubated after a successful SBT-PS (a fortiori if their concomitant SBT-TP test was unsuccessful) might have been underassisted in the post-extubation period, as only 78% of the patients received NIV during 50% of the time (Additional file 13). As a consequence, combining SBT-PS with the systematic use of post-extubation NIV in association with nasal high-flow oxygen to provide continuous post-extubation respiratory assistance might be a valuable strategy to investigate in the future.

The present study had several strengths. First, the study is the first randomized controlled trial comparing daily SBT-TP and SBT-PS in an exclusive population of difficult-to-wean intubated patients who failed their first SBT using a T-piece. Second, the statistical analysis took into account the competitive risk of death regarding the primary outcome criterion. Third, successful extubation was defined by the lack of reintubation on day 7 as opposed to a shorter time frame (i.e., 72 h), avoiding the underestimation of late extubation failure.

However, multiple limitations should be noted. First, the study is underpowered to identify small differences between groups, as a higher effect size was expected during sample size computation. Second, while the global rate of protocol violations was similar in both groups, the extensively-assisted weaning group might have been disadvantaged by a higher rate of NIV use despite not required by the protocol in the standard weaning arm and having a significant proportion of patients who did not receive the additional SBT-TP in the extensively-assisted weaning group, which may have precluded these patients to receive prophylactic NIV. Third, some important baseline characteristics were imbalanced between groups despite randomization, questioning the validity of the results. Fourth, COVID-19 patients represented more than a third of the study population, which may slightly hamper the generalizability of the study results. Fifth, the reintubation rate was significant in our study and may reflect that the included population was different from some large observational studies [19]. Finally, patients reintubated for short medical/surgical procedures were not deemed as extubation failure, although it is unlikely that this has biased the study as these events were uncommon (3 events) and well balanced between arms (1 event in the extensively-assisted weaning group and 2 events in the standard weaning group).

Regarding clinical implications, the present study does not support a preferential SBT modality in patients failing their first SBT, in opposition to the general ICU population in which SBT-PS has been shown to be superior to SBT-TP [5, 26]. Once a patient is identified as difficult-to-wean by failing a first SBT-PS, SBT-TP might, however, be more appropriate to evaluate the patient’s ability to breathe spontaneously in subsequent SBT. On the other hand, if SBT-PS is chosen, the addition of PEEP 5 cmH_2_O to PS during SBT may be harmful in this population, as it may promote anticipated and inadequate extubation and may be associated with a higher rate of extubation failure.

## Conclusion

An extensively-assisted weaning strategy combining SBT-PS with PEEP to assess the ability to breathe spontaneously and SBT-TP to trigger additional indication of post-extubation prophylactic NIV did not lead to a shorter time to successful extubation when compared to a standard weaning strategy using SBT-TP.

### Supplementary Information

**Additional file 1. **Weaning readiness criteria.

**Additional file 2. **Eligibility criteria.

**Additional file 3. **Description of interventions according to allocated group.

**Additional file 4. **Spontaneous breathing trial failure criteria.

**Additional file 5. **Cough and abundancy of respiratory secretions scores.

**Additional file 6. **Readiness to extubate criteria.

**Additional file 7. **Reintubation criteria.

**Additional file 8. **Modification of secondary outcomes and additional post hoc outcomes added during reviewing process.

**Additional file 9. **Risk factors for extubation failure.

**Additional file 10. **Description of *post hoc* analyses.

**Additional file 11. **Cumulative incidence curve for successful extubation or death per group.

**Additional file 12. **Kaplan Meier curve depicting time to first extubation attempt.

**Additional file 13. **Characteristics of all extubation episodes.

**Additional file 14. **Rate of succesful extubation according to study group and spontaneous breathing trial results.

**Additional file 15. **Characteristics of reintubation episodes.

**Additional file 16. **Weaning group according to randomization.

**Additional file 17. **Protocol violations.

## Data Availability

Study datasets are available upon reasonable request to the corresponding author.

## Abbreviations

CHF: Chronic heart failure
CI_95%_: 95% Confidence interval
COPD: Chronic obstructive pulmonary disease
CRF: Chronic respiratory failure
HR: Hazard ratio
ICU: Intensive care unit
NIV: Non-invasive ventilation
PEEP: Positive end-expiratory pressure
PS: Pressure support
SAPS2: Simplified acute physiology score II
SBT: Spontaneous breathing trial
SBT-PS: Spontaneous breathing trial with pressure support
SBT-TP: Spontaneous breathing trial with T-piece
SHR: Subdistribution hazard ratio
WIPO: Weaning induced pulmonary oedema
ZEEP: Zero end-expiratory pressure
