# Supplementary material for: Spontaneous breathing trial with pressure support on positive end-expiratory pressure and extensive use of non-invasive ventilation versus T-piece in difficult-to-wean patients from mechanical ventilation: a randomized controlled trial
Source: Ann Intensive Care. 2024 Apr 17;14:59. doi: 10.1186/s13613-024-01290-6 (PMC11024068; doi:10.1186/s13613-024-01290-6)
Supplement: Supplementary file 1 — Additional file 1. Weaning readiness criteria. [file 13613_2024_1290_MOESM1_ESM.docx]

| **Additional file 1. Weaning readiness criteria*** | |
| --- | --- |
| Hemodynamic criteria | Norepinephrine < 1mg/h  Dobutamine ≤ 5 µg/kg/min |
| Respiratory criteria | FiO_2_ ≤ 50%  SpO_2_ ≥ 88%  PEEP ≤ 5 cmH_2_O  Respiratory rate ≤ 35/min |
| Neurological criteria | Response to verbal orders |

* all criteria must be fulfilled to assess weaning readiness status

FiO_2_ denotes inspired fraction of oxygen; SpO_2_, peripheral oxygen saturation; and PEEP, positive end-expiratory pressure
