# Supplementary material for: Spontaneous breathing trial with pressure support on positive end-expiratory pressure and extensive use of non-invasive ventilation versus T-piece in difficult-to-wean patients from mechanical ventilation: a randomized controlled trial
Source: Ann Intensive Care. 2024 Apr 17;14:59. doi: 10.1186/s13613-024-01290-6 (PMC11024068; doi:10.1186/s13613-024-01290-6)
Supplement: Supplementary file 2 — Additional file 2. Eligibility criteria. [file 13613_2024_1290_MOESM2_ESM.docx]

**Additional file 2. Eligibility criteria**

| Inclusion criteria   - Patient aged 18 years or older - Intubated and ventilated in the intensive care unit for more than 24 hours - Patient with weaning readiness criteria (additional file 1) - Failure of the first SBT-TP   Non-inclusion criteria   - Chronic neuromuscular disease - Guillain-Barré syndrome - Central nervous system disease with consciousness disorder (i.e. inability to respond to verbal orders) - Tracheostomy - Chronic disease with life expectancy less than 1 year - Pregnancy or breastfeeding - Withholding life support regarding a reintubation - Prisoner or patient interned in a psychiatric hospital - Guardianship - Language barrier - Lack of social security - Lack of the patient’s consent (or next of kin when appropriate) - Patient under an exclusion period after enrollment in another research study |
| --- |

SBT-TP denotes spontaneous breathing trial with T-piece.
