# Supplementary material for: Spontaneous breathing trial with pressure support on positive end-expiratory pressure and extensive use of non-invasive ventilation versus T-piece in difficult-to-wean patients from mechanical ventilation: a randomized controlled trial
Source: Ann Intensive Care. 2024 Apr 17;14:59. doi: 10.1186/s13613-024-01290-6 (PMC11024068; doi:10.1186/s13613-024-01290-6)
Supplement: Supplementary file 3 — Additional file 3. Description of interventions according to allocated group. [file 13613_2024_1290_MOESM3_ESM.docx]

**Additional file 3. Description of interventions according to allocated group.**

NIV denotes non-invasive ventilation; SBT-TP, spontaneous breathing trial with T-piece; and SBT-PS with PEEP, spontaneous breathing trial with pressure support 7 cmH_2_O and positive end-expiratory pressure 5 cmH_2_O.

* If readiness to extubate criteria present (see additional file 6)

** Post-extubation NIV criteria were the following: age > 65 yrs, chronic heart or respiratory failure, PaCO_2_ > 45 mmHg during SBT, chronic obstructive pulmonary disease.
