# Supplementary material for: Spontaneous breathing trial with pressure support on positive end-expiratory pressure and extensive use of non-invasive ventilation versus T-piece in difficult-to-wean patients from mechanical ventilation: a randomized controlled trial
Source: Ann Intensive Care. 2024 Apr 17;14:59. doi: 10.1186/s13613-024-01290-6 (PMC11024068; doi:10.1186/s13613-024-01290-6)
Supplement: Supplementary file 4 — Additional file 4. Spontaneous breathing trial failure criteria. [file 13613_2024_1290_MOESM4_ESM.docx]

**Additional file 4. Spontaneous breathing trial failure criteria**

| • Variation of ± 20% of heart rate from pre-SBT value  • Variation of ± 20% of systolic arterial pressure from pre-SBT value  • Respiratory rate > 35/min  • SpO_2_ < 88%  • Sweating, agitation, conscience alteration  • Signs of respiratory distress: increased accessory muscle activity, facial signs of distress, dyspnea  • Arterial pH < 7.35 and PaCO_2_ > 45 mmHg |
| --- |

PaCO_2_ denotes partial pressure of carbon dioxide in arterial blood; SBT, spontaneous breathing trial; and SpO_2_, peripheral oxygen saturation.
