# Supplementary material for: Spontaneous breathing trial with pressure support on positive end-expiratory pressure and extensive use of non-invasive ventilation versus T-piece in difficult-to-wean patients from mechanical ventilation: a randomized controlled trial
Source: Ann Intensive Care. 2024 Apr 17;14:59. doi: 10.1186/s13613-024-01290-6 (PMC11024068; doi:10.1186/s13613-024-01290-6)
Supplement: Supplementary file 5 — Additional file 5. Cough and abundancy of respiratory secretions scores. [file 13613_2024_1290_MOESM5_ESM.docx]

**Additional file 5. Cough and abundancy of respiratory secretions scores**

| Cough strength***** | Abundancy of respiratory secretions** |
| --- | --- |
| 0: no cough on command  1: audible movement of air through the endotracheal tube but no audible cough  2: weak barely audible cough  3: clearly audible cough  4: stronger cough  5: multiple sequential strong coughs | 0 (absent)  1 (low quantity)  2 (intermediate)  3 (abundant)  4 (very abundant) |

*From “Predictors of extubation outcome in patients who have successfully completed a spontaneous breathing trial.” by Khamiees M et al., Chest. 2001;120:1262-1270

**From “Risk Factors for and Prediction by Caregivers of Extubation Failure in ICU Patients.” by Thille AW et al., Critical Care Medicine. 2015;43:613-620
