# Supplementary material for: Spontaneous breathing trial with pressure support on positive end-expiratory pressure and extensive use of non-invasive ventilation versus T-piece in difficult-to-wean patients from mechanical ventilation: a randomized controlled trial
Source: Ann Intensive Care. 2024 Apr 17;14:59. doi: 10.1186/s13613-024-01290-6 (PMC11024068; doi:10.1186/s13613-024-01290-6)
Supplement: Supplementary file 6 — Additional file 6. Readiness to extubate criteria. [file 13613_2024_1290_MOESM6_ESM.docx]

**Additional file 6. Readiness to extubate criteria**

At least 3 of the following criteria had to be fulfilled for readiness to extubate.

| Semi-quantitative cough strength score ≥ 3 |
| --- |
| Abundancy of respiratory secretions score ≤ 2 |
| No planned surgery in the next 24h |
| No suspicion for post-extubation laryngeal oedema * |
| * Suspicion for post-extubation laryngeal oedema was clinically determined by the physician in charge of the patient |
