# Supplementary material for: Spontaneous breathing trial with pressure support on positive end-expiratory pressure and extensive use of non-invasive ventilation versus T-piece in difficult-to-wean patients from mechanical ventilation: a randomized controlled trial
Source: Ann Intensive Care. 2024 Apr 17;14:59. doi: 10.1186/s13613-024-01290-6 (PMC11024068; doi:10.1186/s13613-024-01290-6)
Supplement: Supplementary file 7 — Additional file 7. Reintubation criteria. [file 13613_2024_1290_MOESM7_ESM.docx]

**Additional file 7. Reintubation criteria**

One of the following criteria had to be fulfilled for reintubation.

| 1- respiratory failure defined by occurrence of at least one of the following criteria: respiratory rate > 40/min, signs of respiratory distress, copious respiratory secretions, pH<7.35 with PaCO_2_ > 45 mmHg, SpO_2_ < 90% or PaO_2_ < 60 mmHg under FiO_2_ ≥ 60% or oxygen flow ≥10 L/min |
| --- |
| 2- coma (defined as coma Glasgow scale <8) |
| 3- hemodynamic failure defined as norepinephrine dose>1 mg/h or onset of lactic acidosis |

PaCO_2_ denotes carbon dioxide partial pressure in arterial blood; PaO_2_, oxygen partial pressure in arterial blood; and SpO_2_ peripheral oxygen saturation.
