# Supplementary material for: Spontaneous breathing trial with pressure support on positive end-expiratory pressure and extensive use of non-invasive ventilation versus T-piece in difficult-to-wean patients from mechanical ventilation: a randomized controlled trial
Source: Ann Intensive Care. 2024 Apr 17;14:59. doi: 10.1186/s13613-024-01290-6 (PMC11024068; doi:10.1186/s13613-024-01290-6)
Supplement: Supplementary file 8 — Additional file 8. Modification of secondary outcomes and additional post hoc outcomes added during reviewing process. [file 13613_2024_1290_MOESM8_ESM.docx]

| **Additional file 8. Modification of secondary outcomes and additional *post hoc* outcomes added during reviewing process** | | | |
| --- | --- | --- | --- |
| Outcomes | Original protocol version | Final protocol version | Final version after reviewing process |
| **Secondary outcomes** |  |  | |
| Rate of successful extubation on the first attempt | Percentage (%) of patients with a first successful extubation divided by the total number of *patients extubated* | Percentage (%) of patients with a first successful extubation divided by the total number of *patients per group* | |
| Intensive care unit length of stay | Time (days) spent in intensive care unit from *admission* until discharge or death | Time (days) spent in intensive care unit from *inclusion* until discharge or death | |
| Hospital length of stay | Time (days) spent in the hospital from *admission* until discharge or death | Time (days) spent in the hospital from *inclusion* until discharge or death | |
| Reintubation rate | Percentage (%) of the *number of reintubation divided by the total number of extubation* | Percentage (%) of the *number of patients with any reintubation divided by the number of patients per group* | Percentage (%) of the *number of patients with any reintubation divided by the total number of extubated patients per group* |
| **Additional *post hoc* outcomes added during reviewing process** | | | |
| Rate of successful first SBT after inclusion | Not planned | Not planned | Added during the reviewing process |
| Rate of successful extubation on day-1 after the first SBT | Not planned | Not planned | Added during the reviewing process |
| Rate of successful extubation within 7 days after inclusion | Not planned | Not planned | Added during the reviewing process |
| Rate of patients experiencing an extubation attempt | Not planned | Not planned | Added during the reviewing process |
| Rate of patients with a self extubation | Not planned | Not planned | Added during the reviewing process |
| Minor modifications occurred to these secondary outcomes, after beginning of the trial and before database lock. These modifications were declared to clinical trial.gov and reported in the published version of the protocol (“Pressure support and positive end-expiratory pressure versus T-piece during spontaneous breathing trial in difficult weaning from mechanical ventilation: study protocol for the SBT-ICU study.” by Mezidi et al. Trials. 2022;23:993.). Reintubation rate computation was altered during reviewing process. Additional *post hoc* outcomes were added during reviewing process | | | |
