# Supplementary material for: Spontaneous breathing trial with pressure support on positive end-expiratory pressure and extensive use of non-invasive ventilation versus T-piece in difficult-to-wean patients from mechanical ventilation: a randomized controlled trial
Source: Ann Intensive Care. 2024 Apr 17;14:59. doi: 10.1186/s13613-024-01290-6 (PMC11024068; doi:10.1186/s13613-024-01290-6)
Supplement: Supplementary file 9 — Additional file 9. Risk factors for extubation failure. [file 13613_2024_1290_MOESM9_ESM.docx]

| **Additional file 9. Risk factors for extubation failure** |
| --- |
| 1. Age>65 yrs. 2. Heart failure as the primary indication for mechanical ventilation 3. Moderate-to-severe chronic obstructive pulmonary disease 4. Acute Physiology And Chronic Health Evaluation (APACHE) II score>12 on extubation day 5. Body mass index (weight in kg/height in m^2^)>30 6. Airway patency problems 7. Inability to deal with respiratory secretions 8. Difficult or prolonged weaning (failing≥1 attempt at disconnection from mechanical ventilation) 9. ≥2 comorbidities 10. Mechanical ventilation≥7 days 11. Hypercapnia (PaCO2>45 mmHg) at the end of the spontaneous breathing trial |
| Patients were considered at high-risk of extubation failure if having ≥4 risk factors  From “Effect of postextubation noninvasive ventilation with active humidification vs high-flow nasal cannula on reintubation in patients at very high risk for extubation failure: a randomized trial.” by Hernández G et al. Intensive Care Med. 2022;48:1751-1759. |
