# Supplementary material for: Spontaneous breathing trial with pressure support on positive end-expiratory pressure and extensive use of non-invasive ventilation versus T-piece in difficult-to-wean patients from mechanical ventilation: a randomized controlled trial
Source: Ann Intensive Care. 2024 Apr 17;14:59. doi: 10.1186/s13613-024-01290-6 (PMC11024068; doi:10.1186/s13613-024-01290-6)
Supplement: Supplementary file 10 — Additional file 10. Description of post hoc analyses. [file 13613_2024_1290_MOESM10_ESM.docx]

| **Additional file 10. Description of *post hoc* analyses** | |
| --- | --- |
| Variables | Location |
| Cumulative fluid balance between intensive care unit admission and inclusion | Table 1 |
| Time to first extubation attempt | Results and additional file 12 |
| Characteristics of all extubations episodes | Additional file 13 |
| Percentage of time spent under prophylactic NIV while extubated during day of extubation and following day | Additional file 13 |
| Prevalence of very high-risk of extubation failure | Additional file 13 |
| Rate of successful extubation according to study group and spontaneous breathing trial result | Additional file 14 |
| Weaning group according to randomization | Additional file 16 |
| NIV denotes non-invasive ventilation | |
