# Supplementary material for: Spontaneous breathing trial with pressure support on positive end-expiratory pressure and extensive use of non-invasive ventilation versus T-piece in difficult-to-wean patients from mechanical ventilation: a randomized controlled trial
Source: Ann Intensive Care. 2024 Apr 17;14:59. doi: 10.1186/s13613-024-01290-6 (PMC11024068; doi:10.1186/s13613-024-01290-6)
Supplement: Supplementary file 11 — Additional file 11. Cumulative incidence curve for successful extubation or death per group. [file 13613_2024_1290_MOESM11_ESM.docx]

**Additional file 11. Cumulative incidence curve for successful extubation or death per group**


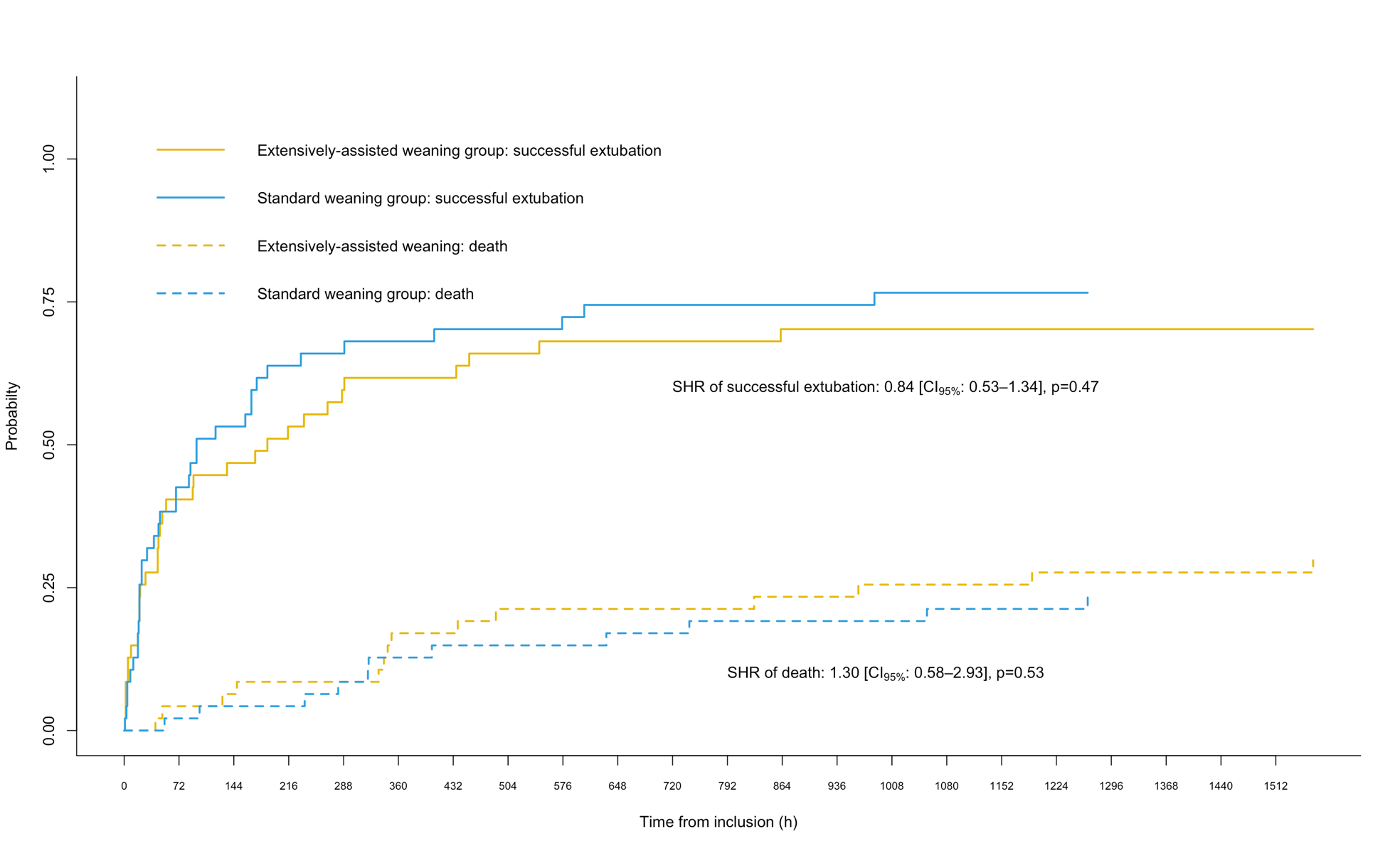


SHR denotes subdistribution hazard ratio; and CI_95%_, 95% confidence interval
