# Supplementary material for: Spontaneous breathing trial with pressure support on positive end-expiratory pressure and extensive use of non-invasive ventilation versus T-piece in difficult-to-wean patients from mechanical ventilation: a randomized controlled trial
Source: Ann Intensive Care. 2024 Apr 17;14:59. doi: 10.1186/s13613-024-01290-6 (PMC11024068; doi:10.1186/s13613-024-01290-6)
Supplement: Supplementary file 12 — Additional file 12. Kaplan Meier curve depicting time to first extubation attempt. [file 13613_2024_1290_MOESM12_ESM.docx]

**Additional Figure 12. Kaplan Meier curve depicting time to first extubation attempt.**


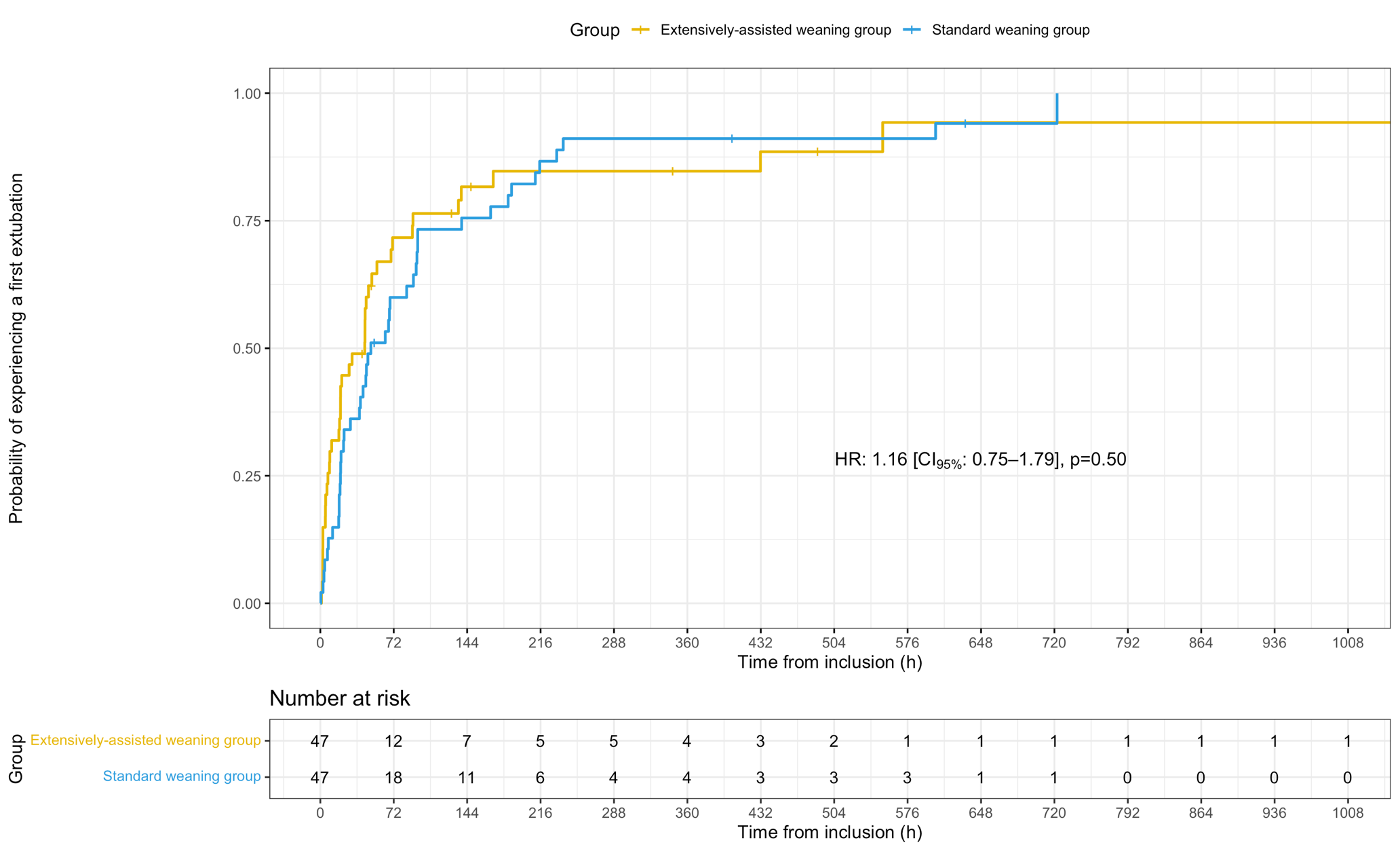


HR denotes hazard ratio, CI_95%_: 95% confidence interval
