# Supplementary material for: Spontaneous breathing trial with pressure support on positive end-expiratory pressure and extensive use of non-invasive ventilation versus T-piece in difficult-to-wean patients from mechanical ventilation: a randomized controlled trial
Source: Ann Intensive Care. 2024 Apr 17;14:59. doi: 10.1186/s13613-024-01290-6 (PMC11024068; doi:10.1186/s13613-024-01290-6)
Supplement: Supplementary file 13 — Additional file 13. Characteristics of all extubation episodes. [file 13613_2024_1290_MOESM13_ESM.docx]

| **Additional file 13. Characteristics of all extubation episodes** | | | | |
| --- | --- | --- | --- | --- |
| Variables | Extensively-assisted weaning group (n=72) | Standard weaning group (n=61) | Absolute difference  [CI_95%_] * | *p-value* |
| Number of patients – no. | 41 | 44 | - |  |
| Additional SBT with T-piece |  |  |  |  |
| Failure – no. (%)** | 18 (25%) | NA | - |  |
| Success – no. (%)** | 34 (47%) | NA | - |  |
| Not done – no. (%)** | 20 (28%) | NA | - |  |
| Presence of ≥ 4 risk factors of extubation failure – no. (%)*** | 65 (90%) | 54 (89%) | 2 [-9–12]% | 0.78 |
| Extubation type |  |  |  | **0.02** |
| Auto-extubation – no. (%) | 12 (17%) | 3 (5%) | 12 [2–22]% |  |
| Planned extubation according to protocol – no. (%) | 60 (83%) | 56 (92%) | -8 [-19–2]% |  |
| Planned extubation despite protocol – no. (%) | 0 (0%) | 2 (3%) | -3 [-8–0]% |  |
| Patients with prophylactic post-extubation NIV – no. (%) | 56 (78%) | 43 (71%) | 7 [-7–23]% | 0.43 |
| Median duration of prophylactic NIV while extubated during day of extubation and following day [IQR] – hrs. § | 9 [3– 14] | 12 [6–20] | -2 [-6–1] | 0.18 |
| Median percentage of time spent on prophylactic NIV while extubated during day of extubation and following day [IQR] – % § | 51 [30–82]% | 47 [31–67]% | 4 [-8–17]% | 0.50 |
| All the analysis of the table were unplanned and *post hoc*.  CI_95%_ denotes 95% confidence interval; FiO_2_: inspired fraction of oxygen; IQR: interquartile range; NA, not applicable; NIV: non-invasive ventilation; PaCO_2_: partial pressure of carbon dioxide; PaO_2_: partial pressure of arterial oxygen; and SBT, spontaneous breathing trial.  * Absolute difference and CI_95%_ were computed for proportions through bootstrapping and for median through Hodges-Lehmann method. CI_95%_ were not corrected for multiple comparisons and should be considered as exploratory.  ** Data available for all episodes but 12 episodes in the extensively-assisted weaning group and 4 in the standard weaning group *** Risk factors for extubation failure were: age>65 yrs., heart failure as the primary indication for mechanical ventilation, moderate-to-severe chronic obstructive pulmonary disease, Acute Physiology And Chronic Health Evaluation (APACHE) II score>12 on extubation day, body mass index (weight in kg/height in m^2^)>30, airway patency problems, inability to deal with respiratory secretions, difficult or prolonged weaning (failing≥1 attempt at disconnection from mechanical ventilation), ≥2 comorbidities, mechanical ventilation≥7 days, and/or hypercapnia (PaCO2>45 mmHg) at the end of the SBT. From “Effect of postextubation noninvasive ventilation with active humidification vs high-flow nasal cannula on reintubation in patients at very high risk for extubation failure: a randomized trial.” by Hernández G et al. Intensive Care Med. 2022;48:1751-1759.  §Among episodes where patients were receiving prophylactic NIV (extensively-assisted weaning group: 56 and the standard weaning group: 43) | | | | |
