# Supplementary material for: Spontaneous breathing trial with pressure support on positive end-expiratory pressure and extensive use of non-invasive ventilation versus T-piece in difficult-to-wean patients from mechanical ventilation: a randomized controlled trial
Source: Ann Intensive Care. 2024 Apr 17;14:59. doi: 10.1186/s13613-024-01290-6 (PMC11024068; doi:10.1186/s13613-024-01290-6)
Supplement: Supplementary file 14 — Additional file 14. Rate of succesful extubation according to study group and spontaneous breathing trial results. [file 13613_2024_1290_MOESM14_ESM.docx]

**Additional file 14. Rate of succesful extubation according to study group and spontaneous breathing trial results**


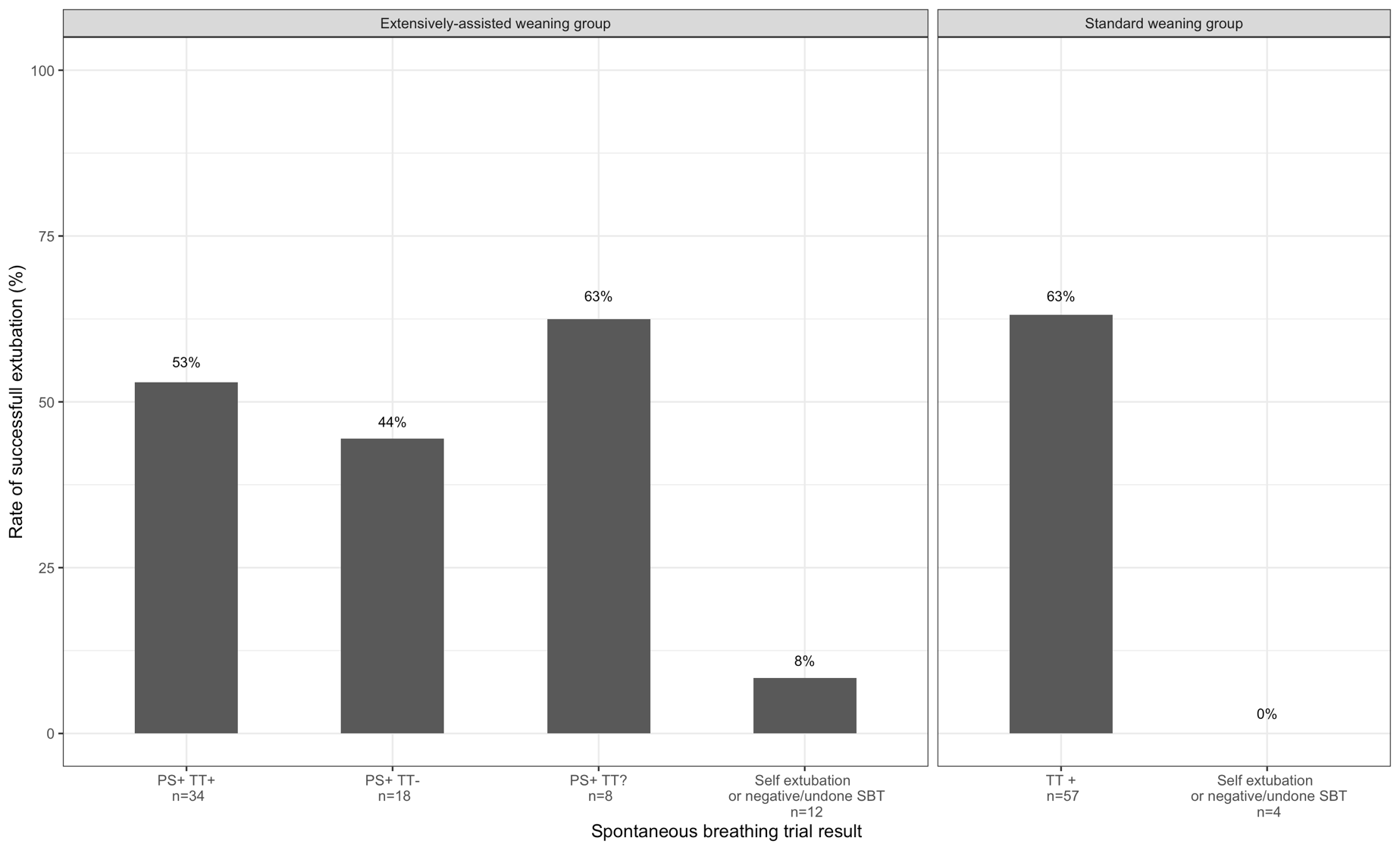


This analysis was unplanned and *post hoc*.

PS+ TT+ denotes success of spontaneous breathing trial (SBT) with pressure support (PS) followed by the success of a SBT with T-piece (TP); PS+ TT- , success of SBT with PS then failure of SBT with TP; PS+ TT? : success of the SBT with PS but SBT with TP not performed; and TT+, success of SBT with TP
