# Supplementary material for: Spontaneous breathing trial with pressure support on positive end-expiratory pressure and extensive use of non-invasive ventilation versus T-piece in difficult-to-wean patients from mechanical ventilation: a randomized controlled trial
Source: Ann Intensive Care. 2024 Apr 17;14:59. doi: 10.1186/s13613-024-01290-6 (PMC11024068; doi:10.1186/s13613-024-01290-6)
Supplement: Supplementary file 15 — Additional file 15. Characteristics of reintubation episodes. [file 13613_2024_1290_MOESM15_ESM.docx]

| **Additional file 15. Characteristics of reintubation episodes** | | | |  |
| --- | --- | --- | --- | --- |
| Variables | Extensively-assisted weaning group (n=39) | Standard weaning group (n=22) | Absolute difference [CI_95%_]* | *p-value* |
| Number of reintubated patients – no. | 18 | 17 | – |  |
| Criteria for reintubation $ |  |  |  |  |
| Hemodynamic criterion – no. (%) | 2 (5%) | 3 (14%) | -9 [-25–6]% | 0.34 |
| Neurological criterion – no. (%) | 4 (10%) | 2 (9%) | 1 [-15–15]% | 1 |
| Respiratory criterion – no. (%) | 36 (92%) | 19 (86%) | 6 [-10–24]% | 0.66 |
| Median time between extubation and reintubation [IQR] – hrs. | 7 [2–49] | 18 [7–61] | -5 [-18–3] | 0.20 |
| CI_95%_ denotes 95% confidence interval; IQR, interquartile range * Absolute difference and CI_95%_ were computed for proportions through bootstrapping, for median through Hodges-Lehmann method. CI_95%_ were not corrected for multiple comparisons and should be considered as exploratory.  $ Multiple criteria could lead to reintubation episodes; hence the sum of criteria is superior to the number of reintubation episodes | | | | |
