# Supplementary material for: Spontaneous breathing trial with pressure support on positive end-expiratory pressure and extensive use of non-invasive ventilation versus T-piece in difficult-to-wean patients from mechanical ventilation: a randomized controlled trial
Source: Ann Intensive Care. 2024 Apr 17;14:59. doi: 10.1186/s13613-024-01290-6 (PMC11024068; doi:10.1186/s13613-024-01290-6)
Supplement: Supplementary file 16 — Additional file 16. Weaning group according to randomization. [file 13613_2024_1290_MOESM16_ESM.docx]

**Additional file 16. Weaning group according to randomization**


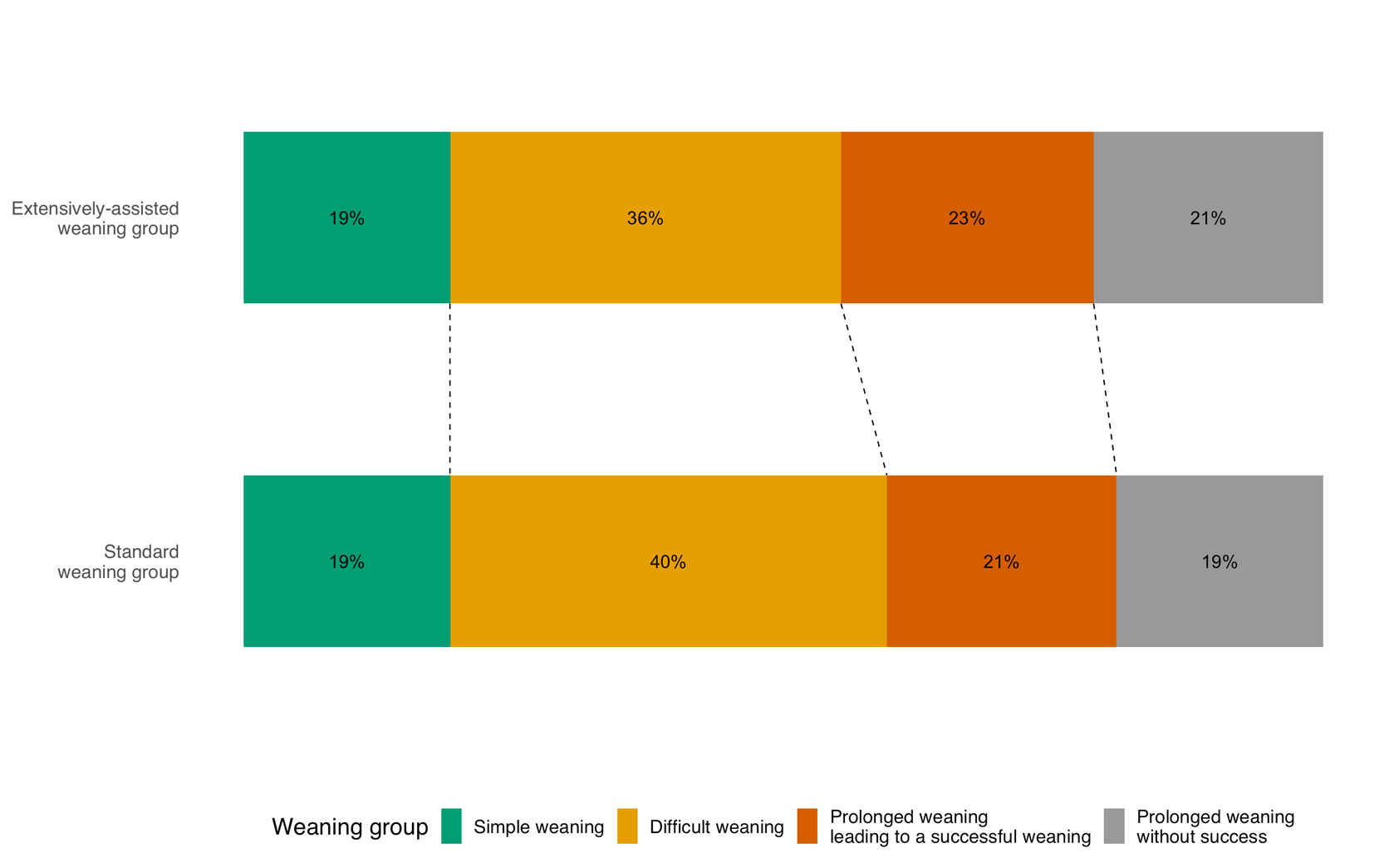


Weaning group were defined according to Béduneau G et al. Epidemiology of weaning outcome according to a new definition. The WIND study. American Journal of Respiratory and Critical Care Medicine, 2017;195:772-783.

Patients classified as simple weaning were either successfully extubated after a first successful SBT on the day of randomization, or on the following day if successful extubation occurred within 24 hours of their first separation attempt from the ventilator.
