# Supplementary material for: Spontaneous breathing trial with pressure support on positive end-expiratory pressure and extensive use of non-invasive ventilation versus T-piece in difficult-to-wean patients from mechanical ventilation: a randomized controlled trial
Source: Ann Intensive Care. 2024 Apr 17;14:59. doi: 10.1186/s13613-024-01290-6 (PMC11024068; doi:10.1186/s13613-024-01290-6)
Supplement: Supplementary file 17 — Additional file 17. Protocol violations. [file 13613_2024_1290_MOESM17_ESM.docx]

**Additional file 17. Protocol violations**

| Variables | Extensively-assisted weaning group (n=47) | Standard weaning group  (n=47) | Absolute difference [CI_95%_]* | *p-value* |
| --- | --- | --- | --- | --- |
| Number of patients with ≥ 1 protocol violation – no. (%) | 18 (38%) | 17 (36%) | 2 [-17–21]% | 1 |
| Patients not extubated despite protocol mandated – no. (%) | 6 (13%) | 7 (15%) | -2 [-15–13]% | 1 |
| Patients extubated despite lack of weaning or extubability criteria – no. (%) | 0 (0%) | 2 (4%) | -4 [-11–0]% | 0.49 |
| Post-extubation NIV performed despite not required per protocol – no. (%) | 1 (2%) | 6 (13%) | -11 [-21–0]% | 0.11 |
| Post-extubation rescue NIV – no. (%) | 1 (2%) | 2 (4%) | -2 [-9–4]% | 1 |
| Post-extubation NIV not done despite protocol mandated – no. (%) | 3 (6%) | 1 (2%) | 4 [-4–13]% | 0.62 |
| Additional SBT-TP not done after SBT-PS success in extensively-assisted weaning group | 13 (28%) | — | — | — |
| CI_95%_ denotes 95% confidence interval; NIV, non-invasive ventilation; and SBT-TP, spontaneous breathing trial with T-piece. * Absolute difference and CI_95%_ were computed for proportions through bootstrapping. CI_95%_ were not corrected for multiple comparisons and should be considered as exploratory. | | | |  |
